# Medical Register of the District of Columbia, for 1867

**Published:** 1867-04

**Authors:** 


Medical Register of the District of Columbia, for 1867. Embracing Notices of the Medical, Benevolent, and Public Institutions of Washington. By J. M. Toner, M.D. Blanchard & Mohun, Washington, D.C. 1867.

We are indebted to the author for a copy of this neatly executed and useful little volume. It is a duodecimo of 102 pages, and contains an amount and variety of information, which makes it interesting and valuable to all members of the profession, whether they ever intend to visit Washington or not. The author has certainly exhibited a remarkable amount of industry and skill in selecting and compressing into so small a compass so large a quantity of important and curious historical and statistical information. He deserves the thanks of the profession and a liberal patronage.

